# Renin–Angiotensin System Antagonism Protects the Diabetic Heart from Ischemia/Reperfusion Injury in Variable Hyperglycemia Duration Settings by a Glucose Transporter Type 4-Mediated Pathway

**DOI:** 10.3390/ph16020238

**Published:** 2023-02-03

**Authors:** Aisha Al-Kouh, Fawzi Babiker, Maie Al-Bader

**Affiliations:** Department of Physiology, Faculty of Medicine, Kuwait University, P.O. Box 24923, Kuwait City 13110, Kuwait

**Keywords:** ischemia reperfusion, RAS, captopril, losartan, diabetes mellitus, hyperglycemia

## Abstract

Background: Diabetes mellitus (DM) is a risk factor for cardiovascular diseases, specifically, the ischemic heart diseases (IHD). The renin–angiotensin system (RAS) affects the heart directly and indirectly. However, its role in the protection of the heart against I/R injury is not completely understood. The aim of the current study was to evaluate the efficacy of the angiotensin-converting enzyme (ACE) inhibitor and Angiotensin II receptor (AT1R) blocker or a combination thereof in protection of the heart from I/R injury. Methods: Hearts isolated from adult male Wistar rats (*n* = 8) were subjected to high glucose levels; acute hyperglycemia or streptozotocin (STZ)-induced diabetes were used in this study. Hearts were subjected to I/R injury, treated with Captopril, an ACE inhibitor; Losartan, an AT1R antagonist; or a combination thereof. Hemodynamics data were measured using a suitable software for that purpose. Additionally, infarct size was evaluated using 2,3,5-Triphenyltetrazolium chloride (TTC) staining. The levels of apoptosis markers (caspase-3 and -8), antioxidant enzymes, superoxide dismutase (SOD) and catalase (CAT), nitric oxide synthase (eNOS), and glucose transporter type 4 (GLUT-4) protein levels were evaluated by Western blotting. Pro-inflammatory and anti-inflammatory cytokines levels were evaluated by enzyme-linked immunosorbent assay (ELISA). Results: Captopril and Losartan alone or in combination abolished the effect of I/R injury in hearts subjected to acute hyperglycemia or STZ-induced diabetes. There was a significant (*p* < 0.05) recovery in hemodynamics, infarct size, and apoptosis markers following the treatment with Captopril, Losartan, or their combination. Treatment with Captopril, Losartan, or their combination significantly (*p* < 0.05) reduced pro-inflammatory cytokines and increased GLUT-4 protein levels. Conclusions: The blockade of the RAS system protected the diabetic heart from I/R injury. This protection followed a pathway that utilizes GLUT-4 to decrease the apoptosis markers, pro-inflammatory cytokines, and to increase the anti-inflammatory cytokines. This protection seems to employ a pathway which is not involving ERK1/2 and eNOS.

## 1. Introduction

Cardiovascular diseases (CVDs) are among the leading causes of morbidity and mortality worldwide [1]. Recent studies have reported ischemic heart disease (IHD) as the worldwide leading cause of disability and death [2]. The ultimate effect of ischemia is myocardial infarction (MI), which results from either partial or complete lack of oxygen and nutrients supply to the myocardium [3]. The myocardial reperfusion procedure is the only contemporary standard treatment for the protection of the heart against ischemia/reperfusion (I/R) injury. Nevertheless, development of complications such as in-hospital deaths, reoccurrence of myocardial infarction, and left ventricular (LV) reconditioning leading to heart failure following infarction still exist [4]. Several treatment interventions to prevent myocardial I/R injury have been investigated. Regrettably, the translation of such cardioprotective procedures to the clinical trials did not produce the expected outcome [5,6]. However, these studies were well-planned postconditioning clinical studies, and they resulted in negative results which did not support the previous preclinical findings. Therefore, an urgent need for more effective regimens persists.

Despite the availability of optimal therapy, IHD and its consequences remain high in diabetic patients [7]. Globally, the occurrence of cardiovascular disease in the diabetic population is far greater compared to the nondiabetic population [8]. Furthermore, it was reported that signaling mechanisms of protection from ischemic injury are impaired in chronic diabetes mellitus (DM) [9]; therefore, the threshold for protection of the diabetic heart is significantly increased to a limit that may not be achieved by the traditional procedures [10]. Alteration in signaling pathways, such as survivor activating factor enhancement (SAFE) [11] and reperfusion injury salvage kinase (RISK) [12] is well-established in diabetes, necessitating tailoring new potential therapies for this disease. The effect of hyperglycemia on the outcome of ischemic heart disease is unclear [13]. Coexistence of acute hyperglycemia at occurrence of ischemia may worsen the prognosis of diabetic and nondiabetic patients [14]. The alteration of signaling pathways by diabetes leads to the decrease of major glucose transporter in the heart, glucose transporter 4 (GLUT-4) [15], leading eventually to cardiomyocyte death [16]. Therefore, evaluation of GLUT-1 and GLUT-4 levels will be crucial for understanding the protection of the diabetic heart from I/R injury.

The renin–angiotensin system (RAS) plays a pivotal role in combating myocardial diseases and might ultimately participate in the aggravation of reperfusion injury [17]. Although RASblockers are beneficial during myocardial ischemia, its effects on the diabetic myocardium remain controversial [18,19]. Losartan is a selective antagonist of type 1 angiotensin II receptors (AT1R) and has been used in medical treatments of a variety of cardiovascular diseases [20]. Losartan has been reported to prevent I/R-induced cardiac injury by inhibiting reactive oxygen species (ROS)-induced injury [21]. However, the studies of the effect of Losartan on the diabetic heart remain unresolved and require more clarification. On the other hand, using Captopril, a nonspecific ACE inhibitor, administration resulted in protection of myocardial tissue after I/R insult by preventing left ventricular hypertrophy [22]. We have previously reported the involvement of ACE inhibitors in the protection of the isolated rat heart [23] from the detrimental effect of the locally produced ACE [24,25]. Although dual therapy of RAS antagonism was proven to be protective to the normotensive heart [26,27] and four-week diabetic rats [28], its role in acute hyperglycemia and chronic diabetes was not investigated. Inhibiting angiotensin activity in diabetes by Captopril and Losartan showed great improvement of heart hemodynamics after I/R injury [28]. These regimens could be promising treatment procedures for the protection of the diabetic heart against I/R injury. We aimed in this study to investigate the potential protective effects of Losartan and Captopril and their combination in the protection of heart subjected to acute or chronic hyperglycemia which were known to cause variable pathological effects on the heart.

Chronic inflammation [29,30] and oxidative stress [31] that persist after myocardial infarction were reported to be key mediators of cardiac remodeling. Chronic inflammation interferes metabolically with the heart, indirectly as it jeopardizes cardiac function [32], or directly by interference of the cytokines with calcium transport [33]. Indeed, ROS are crucial for normal cellular function at low levels; however, they were recognized to damage the mitochondria when they reach high concentration levels [18,34,35]. Understanding the effects of inflammation, oxidative stress, and the RAS system in cardiac remodeling in nondiabetic and diabetic hearts will help in developing effective procedures which may produce a better protection to the heart against deterioration from IHD to heart failure.

## 2. Results

In this study, the role of RAS system in the protection of the diabetic heart from I/R injury was investigated. Left ventricular dynamics were assessed throughout the experiment by evaluating left ventricular pressure (LVEDP), LV maximum developed pressure (DPmax), and LV contractility index (±dP/dt). The coronary vascular dynamics were assessed by evaluating coronary flow (CF) and coronary vascular resistance (CVR). There were no significant differences detected in ventricular dynamics, contractility, and coronary vascular dynamics between the groups when at baseline levels. Body and LV weights were not significantly different between the experimental groups (data not shown). Ischemia resulted in a remarkable worsening in the heart functions.

**Figure 1:**
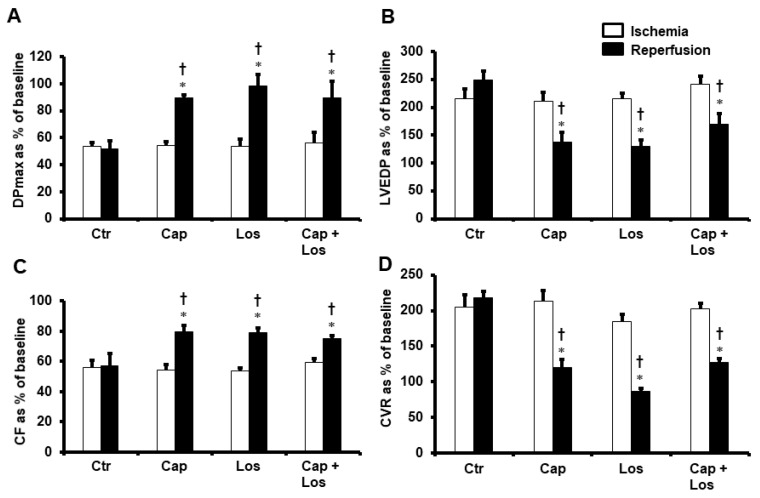

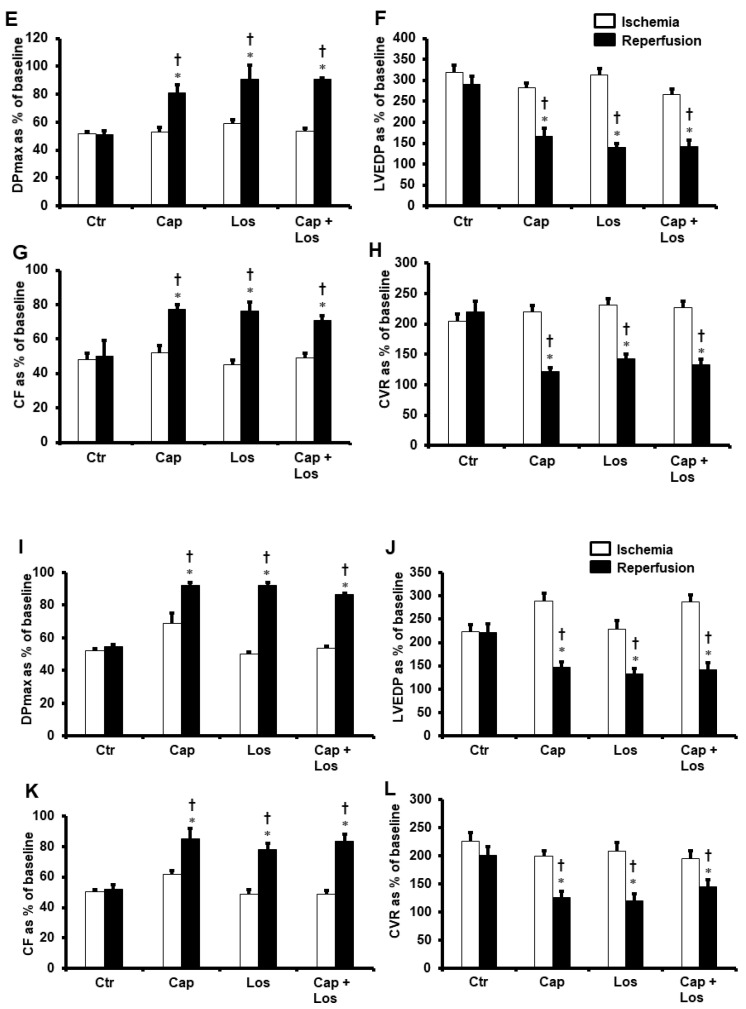
Left ventricle (DPmax and LVEDP) and coronary vascular dynamics (CF and CVR), after DM induction and Captopril and Losartan treatments. (**A**–**D**), hyperglycemia; (**E**–**H**), four weeks diabetes; (**I**–**L**) six weeks diabetes (*n* = 8 per group). The data are presented as the mean ± SEM. DPmax, maximum developed pressure; LVEDP, left ventricular end-diastolic pressure; CF, coronary flow; CVR, coronary vascular resistance; Ctr, control; Cap, Captopril; Los, Losartan; and DM, Diabetes mellitus. * *p* < 0.05 compared to respective controls and † *p* < 0.05 compared to the respective ischemic period.

Captopril, Losartan, or a combination thereof were administered at reperfusion in hearts subjected to hyperglycemia, four weeks, or six weeks of diabetes. These treatments significantly (*p* < 0.05) improved cardiac hemodynamics and vascular dynamics in all three different conditions (hyperglycemia, four weeks, and six weeks diabetic hearts) compared to untreated controls (Figure 1A–C, Table 1). Interestingly, there were no differences in the effects of the three experimental conditions on the protection of the heart, and the combination of the drugs did not show additive effects in all treatments compared to untreated controls (Figure 1, Table 1).

**Figure 2:**
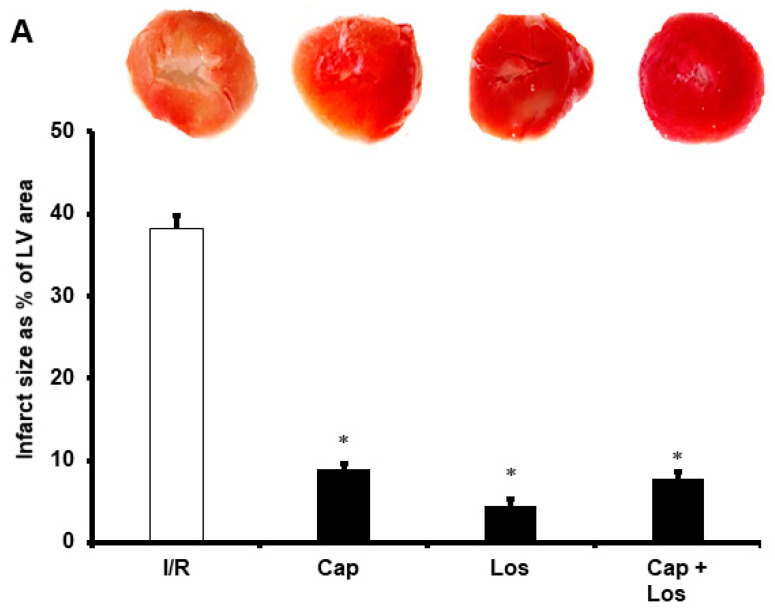

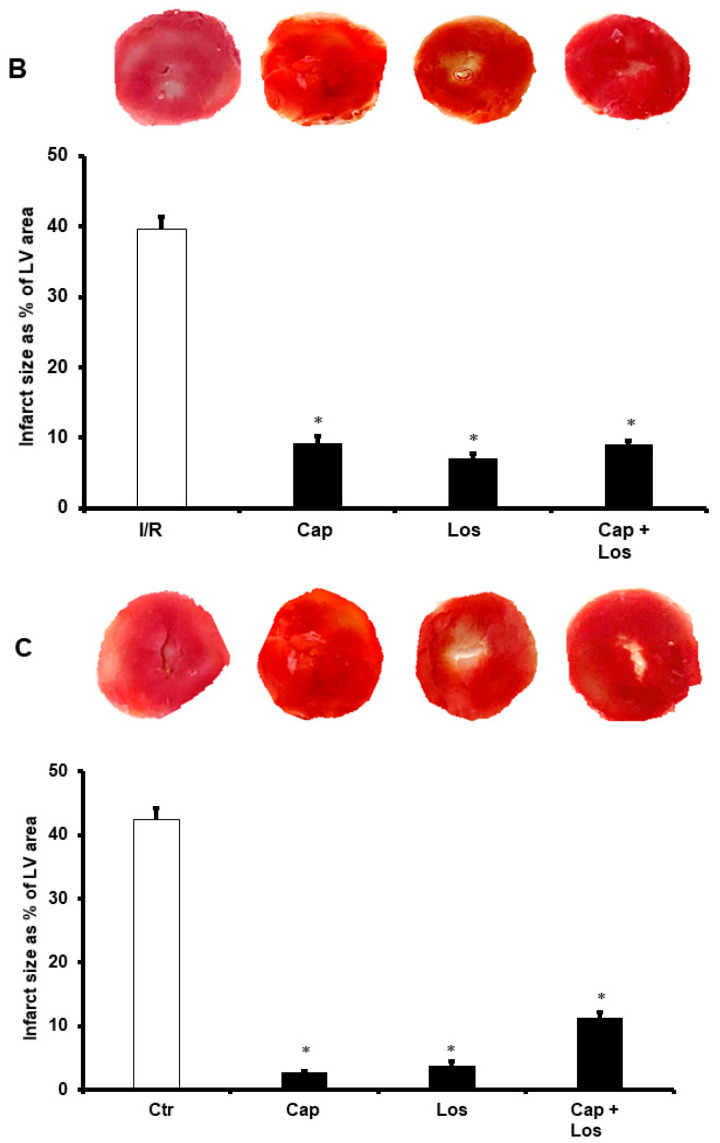
TTC staining assessment of infarct size (*n* = 4 per group). Infarct size presented area as a percentage of left ventricle area in the experimental models of hyperglycemia and DM. (**A**), hyperglycemia; (**B**), four weeks diabetic hearts; (**C**), six weeks diabetic hearts. Ctr, control; Cap, Captopril; Los, Losartan; and DM, Diabetes mellitus. * *p* < 0.001 compared to the respective controls.

**Figure 3:**
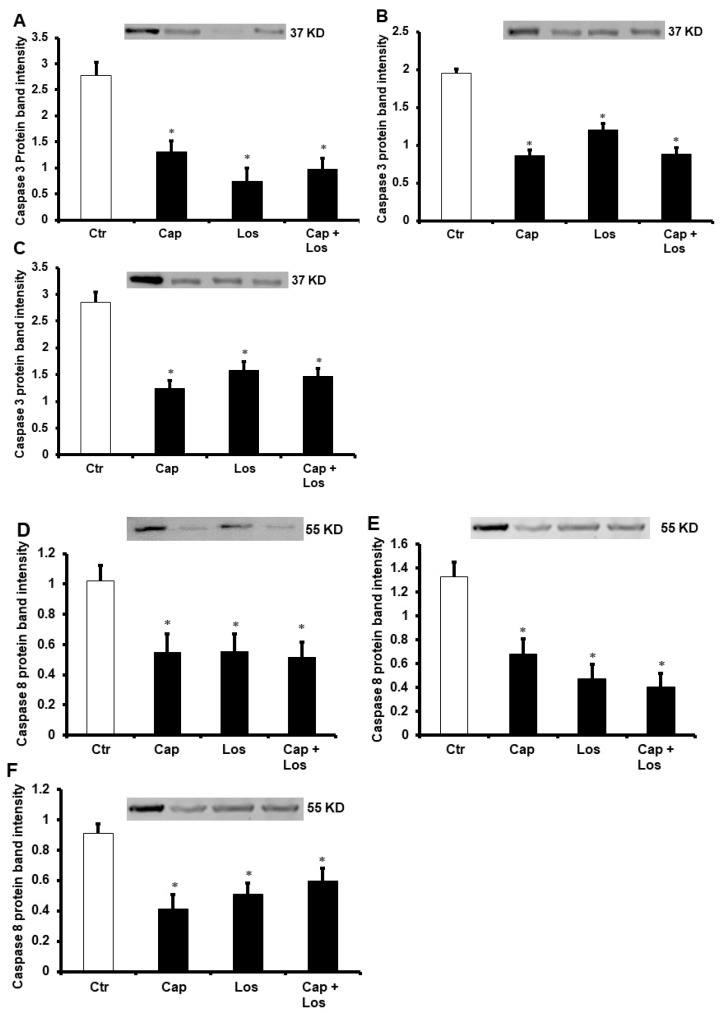
Immunoblotting showing the effects of Captopril, Losartan, or their combination on caspase-3 and caspase-8 protein levels in the left ventricle homogenate (*n* = 4 per group). Western blot evaluations showing the protein levels of caspase-3 (**A**–**C**) and caspase-8 (**D**–**F**). (**A**,**D**), hyperglycemia; (**B**,**E**), four weeks diabetic hearts; (**C**,**F**), six weeks diabetic hearts. Values are presented as means ± SEM. Ctr, control; Cap, Captopril; and Los, Losartan. * *p* < 0.01 compared to respective controls.

Hearts subjected to hyperglycemia, four weeks, or six weeks of diabetes were protected from I/R injury by Captopril, Losartan, or their combination. These drugs resulted in a significant decrease in the infarct size (*p* < 0.001) and cardiac TnT levels. There were no significant differences in the effects of the three experimental conditions on the protection of the heart, and the combination of the drugs did not show additive effects in all treatments compared to untreated controls (*p* < 0.01) (Figure 2 and Table 2). These results were confirmed by a decrease of apoptosis in the myocytes by Captopril and Losartan or their combination. Administration of these drugs at reperfusion resulted in a marked decrease in apoptosis markers caspase-3 and caspase-8 (*p* < 0.01) compared to untreated controls (Figure 3). 

**Figure 4:**
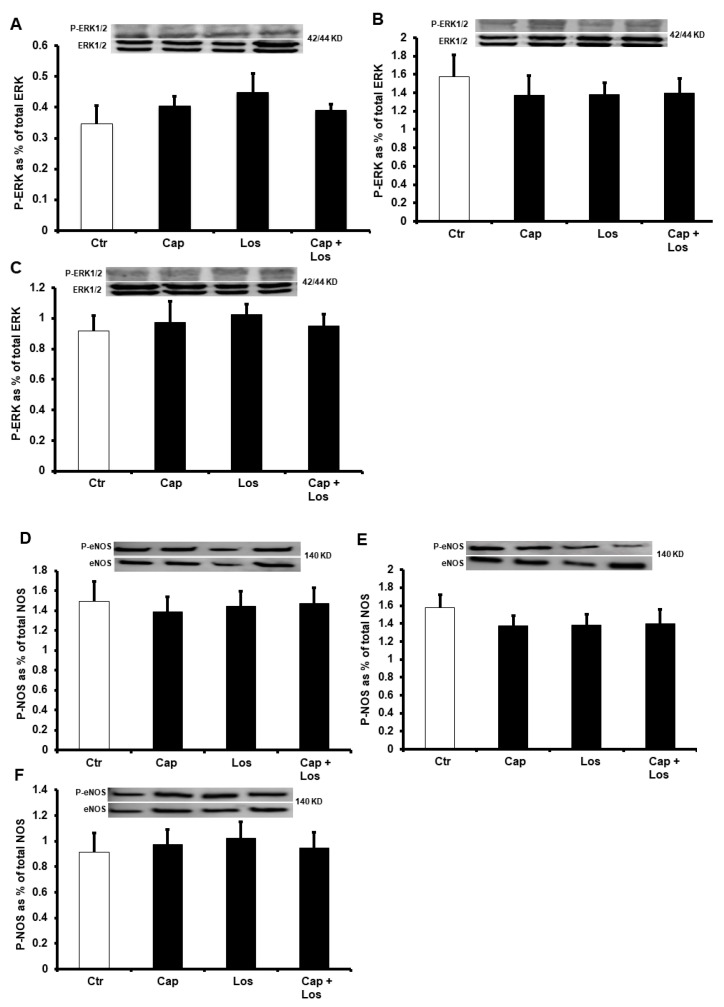
Immunoblotting showing the effects of Captopril, Losartan, or their combination on ERK1/2 and eNOS phosphorylation levels in the left ventricle homogenate (*n* = 4 per group). Western blot evaluations showing ERK1/2 and Phosphorylated ERK1/2 (P-ERK1/2) (**A**–**C**), eNOS and Phosphorylated eNOS (P-eNOS) (**D**–**F**). (**A**,**D**), hyperglycemia; (**B**,**E**), four weeks diabetic hearts; (**C**,**F**), six weeks diabetic hearts. Values are presented as means ± SEM. Ctr, control; Cap, Captopril; Los, Losartan; ERK, extracellular signal-regulated protein kinase; and eNOS, nitric oxide synthase.

**Figure 5:**
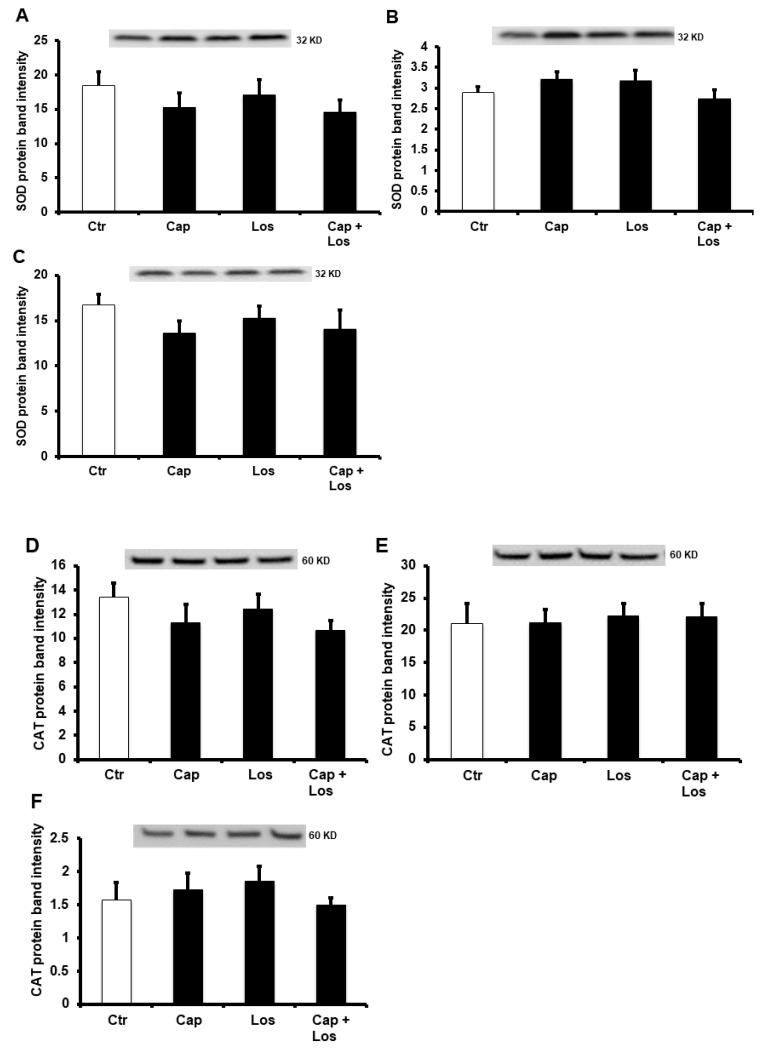
Immunoblotting showing the effects of Captopril, Losartan, or their combination on SOD and CAT protein levels in the left ventricle homogenate (*n* = 4 per group). Immunoblots showing the levels of SOD (**A**–**C**), CAT (**D**–**F**). (**A**,**D**), hyperglycemia; (**B**,**E**), four weeks diabetic hearts; (**C**,**F**), six weeks diabetic hearts. Values are presented as means ± SEM. Ctr, control; Cap, Captopril; Los, Losartan; SOD, superoxide dismutase; and CAT, catalase.

We further evaluated the possible downstream signaling pathways that could be involved in the protection by Captopril, Losartan, or their combination, to the diabetic heart. We tested the effect of these treatments on the protein levels and basal ratio of phosphorylated to total extracellular signal-regulated protein kinase (ERK1/2) or endothelial nitric oxide synthase (eNOS) during I/R injury. Surprisingly, neither ERK1/2 nor eNOS were affected by these treatments. Both protein levels did not show a significant difference between the diabetic hearts and the untreated controls (Figure 4). Surprisingly, the protein levels of superoxide dismutase (SOD) and catalase (CAT) did not show a significant increase in the presence of Captopril, Losartan, or their combination in all the three treatment conditions. (Figure 5). There were no differences in the effects of the three experimental conditions on the protection of the heart, and the combination of the drugs did not show additive effects in all treatments compared to untreated controls (Figure 4 and Figure 5).

**Figure 6:**
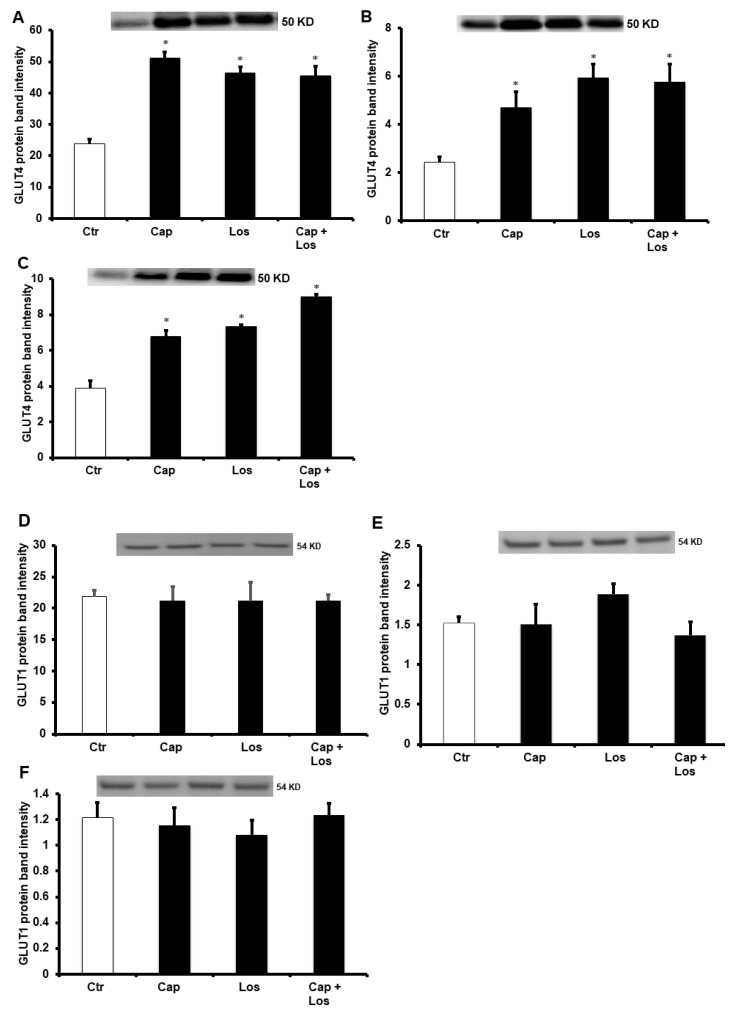
Immunoblotting showing the effects of Captopril and Losartan effects on GLUT-1 and GLUT-4 protein levels in the left ventricle homogenate (*n* = 4 per group). Immunoblots showing the levels of GLUT-4 (**A**–**C**), GLUT-1 (**D**–**F**). (**A**,**D**), hyperglycemia; (**B**,**E**), four weeks diabetic hearts; (**C**,**F**), six weeks diabetic hearts. Values are presented as means ± SEM. Ctr, control; Cap, Captopril; Los, Losartan; and GLU-T, glucose transporter. * *p* < 0.01 compared to the respective controls.

To identify a potential role for glucose transporters, glucose transporter 1 (GLUT-1) and glucose transporter 4 (GLUT-4) protein levels were evaluated using Western blotting. Interestingly, GLUT-4 protein levels were significantly (*p* < 0.01) increased with the treatment of Losartan, Captopril, and their combination, However, no changes in GLUT-1 protein levels were observed. There were no differences in the effects of the three experimental conditions on the protection of the heart, and the combination of the drugs did not show additive effects in all treatments compared to untreated controls (Figure 6).

**Figure 7:**
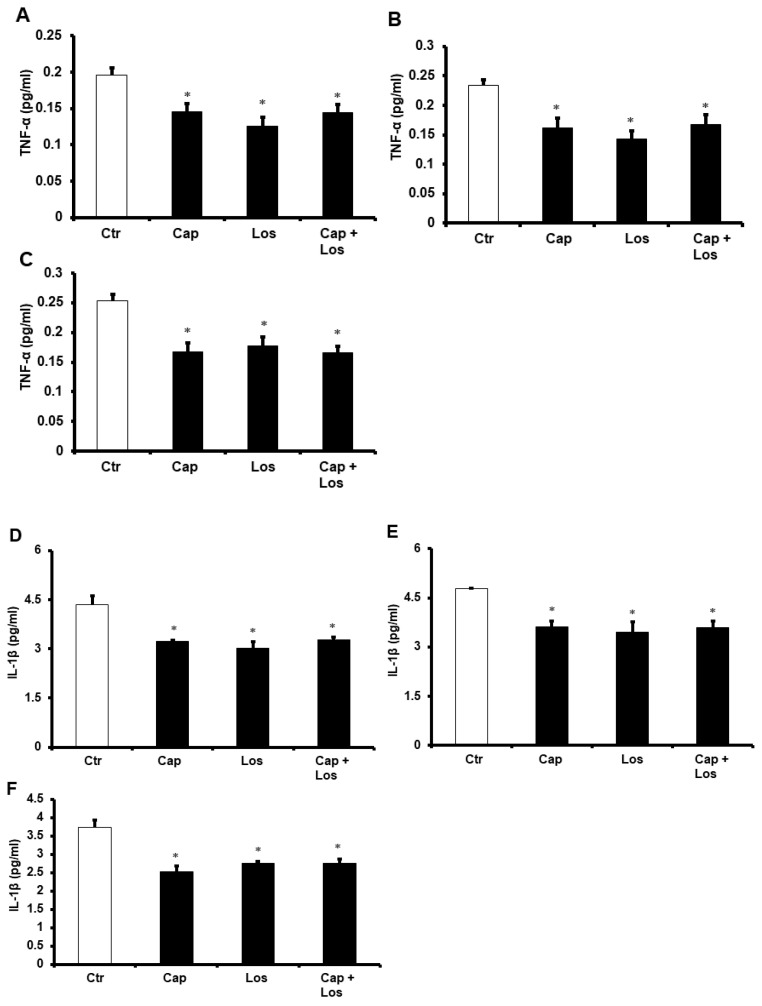

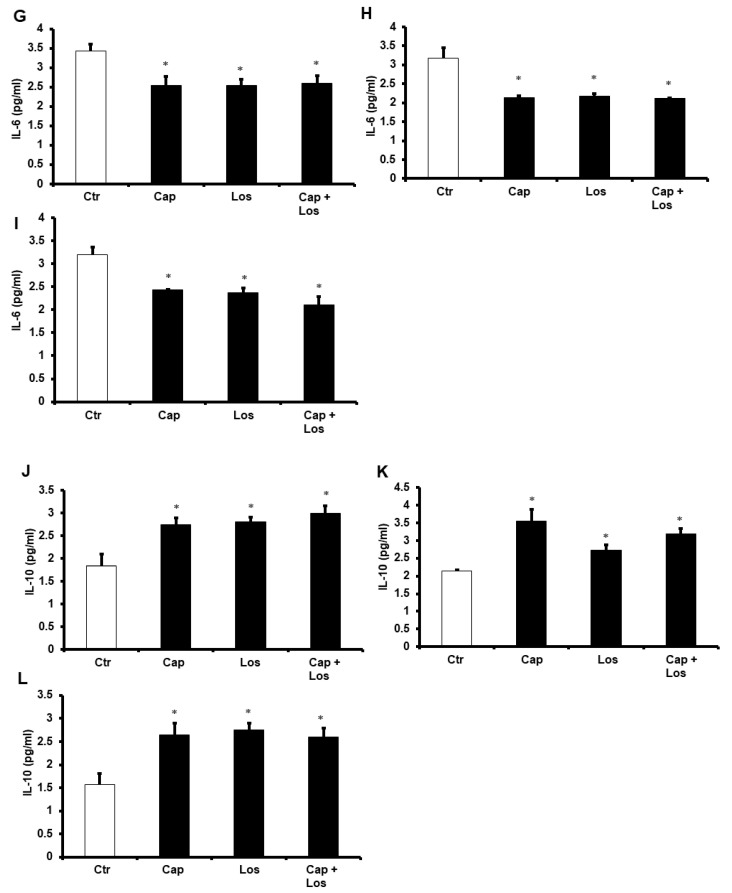
ELISA analysis showing the cytokine levels in the cardiomyocytes after Captopril, Losartan, and the combination of Cap + Los treatment of hearts isolated from four or six weeks compared with those in the control group (*n* = 4 per group). Captopril and Losartan alone or in combination decreased the TNF-α protein levels (**A**–**C**), decreased the IL-1β protein levels (**D**–**F**), decreased the IL-6 protein levels (**G**–**I**), and increased the anti-inflammatory cytokine IL-10 (**J**–**L**) protein levels. (**A**,**D**,**G**,**J**), hyperglycemia; (**B**,**E**,**H**,**K**), four weeks diabetes; (**C**,**F**,**I**,**L**), six weeks diabetes. Ctr, control; Cap, Captopril; Los, Losartan; TNF-α, tumor necrosis factor alpha; IL-1β, interleukin 1 beta; IL-6, interleukin 6; and IL-10, interleukin 10. * *p* < 0.05 compared to the respective controls.

To study the effects of cytokines in the protection of the diabetic heart against I/R injury, we evaluated the levels of the pro-inflammatory cytokines tumor necrosis factor alpha (TNF-α), interleukin 1 beta (IL-1β), and interleukin 6 (IL-6), and anti-inflammatory cytokine interleukin 10 (IL-10) in the cardiomyocyte lysate by enzyme-linked immunosorbent assay (ELISA). Administration of Captopril, Losartan, or their combination resulted in a significant (*p* < 0.01) decrease in TNF-α, IL-1β, and IL-6 levels which was increased at the beginning by ischemia compared to untreated controls (Figure 7A–I). The same treatment resulted in a significant (*p* < 0.05) increase in the anti-inflammatory cytokine IL-10. There were no differences in the effects of the three experimental conditions on the protection of the heart, and the combination of the drugs did not show additive effects in all treatments compared to untreated controls (Figure 7J–L).

## 3. Discussion

The studies of the effects of I/R and its treatments on the diabetic heart are inconsistent. The major area of controversy resides in the dependence of the efficiency of the heart protection on the duration of diabetes before the intervention. Some laboratories consider four weeks of diabetes satisfactory for the pathological effects of diabetes to appear in the heart, and thereafter the animals will be suitable models for the studies. Other laboratories consider this period rather short for the pathology of diabetes to occur and the animal models are similar to normoglycemic animals. Therefore, acute hyperglycemia, four-, and six-week diabetic (chronic hyperglycemia) rats were used in this study to assess the protection of the heart against I/R injury. Although RAS antagonism was reported to be protective to the heart by many investigators, its role in the protection of the diabatic heart is controversial [23,36,37]. The diabetic heart was reported by many researchers to be resistant to protection against I/R injury [10,38]. This alteration in the effect of postconditioning is a consequence of the remodeling caused by the diabetes which blocks or impairs activation of many kinases including, among others, PI3 kinase/Akt, ERK, p70S6 kinase, and/or GSK-3β [39]. However, the data available to date did not reveal significant variations in the injury of the diabetic heart relative to the normotensive heart [23,40,41].

The present study investigated the prospective protection of heart compromised by hyperglycemia and DM (diabetic heart) against I/R injury. The study used hearts subjected to high glucose levels to mimic acute hyperglycemia and diabetic hearts isolated from four- and six-weeks diabetic rats treated at reperfusion with RAS member antagonists (ACE and AT1R). The choice of these conditions was made mainly to understand the potential protection of the diabetic heart in different scenarios with different durations of hyperglycemia. The animal models used for the study of heart protection from I/R injury showed contradicting results when extrapolated to the trials of the protection of the diabetic heart. Some studies reported a notable protection to the diabetic heart [27,42]; however, some other studies reported lack of protection [40,43]. This controversy is evident even in the studies reported by the same laboratory [28,40]. However, substantial uncertainty persisted as two weeks or four weeks are considered the minimum required duration for the diabetic heart-healing disturbance to manifest [44]. Indeed, hyperglycemia even for a short period could raise the threshold for the protection of the diabetic heart [45]; however, some studies reported protection of the four-week diabetic heart against I/R injury [28]. Therefore, this study investigated protection of hearts subjected to different hyperglycemia durations including acute hyperglycemia, four-, and six-week diabetic hearts, to understand the effect of the hyperglycemia duration on the protection, the response of the diabetic heart to the available heart protection procedures, and to find a suitable regimen for the protection of the diabetic heart.

**Figure 8:**
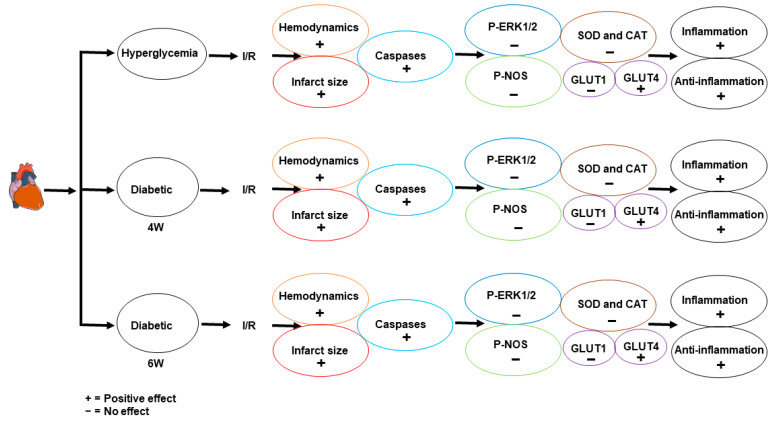
Schematic representation showing the protective effects of Captopril, Losartan, or their combination observed in this study. The arrows in the figure indicate the sequence of the parameter evaluated in the study and not protective pathways. Ctr, control; I/R, ischemia/reperfusion; Phos., Phosphorylation; SOD, Superoxide dismutase; CAT, Catalase; GLUT, Glucose transporter; 4 W, four-week diabetes; and 6 W, six-week diabetes.

This study demonstrates that the diabetic heart could be protected from ischemic injury by RAS antagonism. Captopril, Losartan, or a combination thereof protected the diabetic heart against I/R injury by a signaling pathway that is not utilizing ERK1/2 or eNOS. This protection targets mainly the apoptotic enzymes and pro-inflammatory and anti-inflammatory cytokines (Figure 8). However, although the combination of Captopril and Losartan was protective to the diabetic heart, it does not show additivity in its protection. The diabetic heart was reported previously to be resistant to protection from I/R injury [10]. That could be due to the permanent tissue and organ remodeling induced by diabetes which deteriorates the protective machinery of the classical protective pathways such as RISK and SAFE [9]. In contrast, a lack of blockade for the protection of the nondiabetic heart by the antagonism of AT1R was reported [43]. Nevertheless, controversy in the protection of the diabetic heart still exists. Protection by Losartan was reported in diabetic hearts; however the duration of diabetes was four weeks in these rats [42]. We and others also reported protection of four-week diabetic hearts by ACE and AT1R antagonism [28,42]. In addition, Shi-Ting et al. [46] also showed that four-week diabetic mice have a tolerance to I/R injury which supports the notion that four-week diabetic rats are not fully affected by diabetes. However, this tolerance to ischemia and ease of protection is lacking in six- and eight-week diabetic rats. Combined therapy using ACE inhibitors and AT1R blockers, though not additive, was also reported to be protective to the nondiabetic heart [47]. These notions support the finding of this study as Captopril, Losartan, and their combination protected the heart subjected to hyperglycemia, and four- and six-week diabetic hearts.

To investigate the potentials of this regimen in the treatment of long duration hyperglycemia we used six-week diabetic hearts. Captopril, Losartan, or their combination protected the six-week diabetic hearts as well (Figure 8). In contrast to six- and eight-week diabetic rats, protection of four-week diabetic rats was reported [39]. This indicates the prevalence of permanent pathological changes in six- and eight-week diabetic rats [39]. The intolerance and lack of protection to the diabetic hearts of a long diabetes duration was confirmed by Drenger et al. [11]. These notions indicate an inverse relation between increased diabetic duration and the possibility of heart protection. This study proved the possibility of protection of six-week diabetic hearts from I/R injury. This denotes that although the protective signaling pathways for the protection of the six-week diabetic hearts are defective, some treatment regimen could be protective to these hearts. This protection might have followed a pathway different from the pathways which were previously proven to be effected. The protection reported in the present study seems to follow a pathway using the GLUT-4 receptor.

This study showed that the protection given by Losartan, Captopril, or their combination is through a pathway; however, it is not employing antioxidant enzymes but targeting the pro-inflammatory cytokines (Figure 8). Our results are in line with reports from other laboratories that reported no increase in SOD or CAT activity in the protection of the nondiabetic heart from I/R injury [48,49]. However, although there is a lack of reporting in the literature regarding the effect of RAS antagonism in diabetic animals, we anticipate the lack of increase of the antioxidant enzymes levels in the protection of the diabetic heart is due to the impairment of the AKT/ERK1/2/Nrf2 pathway. The lack of increase in SOD and CAT protein levels in this study could be because of the nature of the RAS antagonists used. The ACE inhibitors and AT1 antagonist blocks the activation of oxidant enzymes and redox-sensitive genes, which may spare the antioxidant enzymes and blunt their increase [50,51]. A lack of synchronous increase in antioxidant enzyme levels with an increased ROS level was reported previously [52]. Furthermore, the protection reported in this study may be following a novel pathway using GLUT-4 in the protection of the diabetic heart. On the other hand, the regulation of the pro-inflammatory cytokines is crucial for the protection of the ischemic heart. Lowering IL-1 and IL-6 availability is associated with reduced heart injury and cardiomyocyte death [53]. Reduced protein levels of TNF-α, IL-1β, and IL-6 presented in this study are similar to those reported by Liu et al. [54]. These notions indicate that RAS antagonism protect the heart by a pathway targeting the pro-inflammatory cytokines. On the other hand, the increased expression of IL-10 in the protection of the diabetic hearts seen in this study was reported before [55].

This study showed an increase in GLUT-4 protein levels (Figure 6). This increase was reported before in the studies of heart protection from I/R injury [56]. The protection reported in the recent study could be due to increased expression of GLUT-4 and improved glucose uptake. GLUT-4 expression was also reported in obese rats with high glucose levels [57]. Similar results that showed increased GLUT-4 but not GLUT-1 levels were reported in the protection of the diabetic heart [58]. A potential limitation of this study is that we did not show the changes in the levels of the cleaved caspases which could give a better indication of apoptosis compared to the levels of the procaspases.

## 4. Materials and Methods

### 4.1. Ischemia Reperfusion Study

Wistar rats of the same age weighing between 270–300 g (*n* = 8 per group) were used in this study. Animal use and treatment were done in agreement with the guidelines of Animal Resource Centre of Health Science Centre, Kuwait University, Kuwait. Rats were housed at 22 °C on a 12 h light/dark cycle (7 am–7 pm), water and food were unrestrictedly available. Anesthesia was performed with intraperitoneal injections of sodium pentobarbital (60 mg/kg) and the animals were anticoagulated by heparin (1000 U/kg). Heart mounting cannulation and perfusion were done as described previously [59,60]. Briefly, isolated heart was reversely perfused with a freshly prepared Krebs–Hensleit (KH) buffer, pH 7.35 to 7.45 at 37.0 ± 0.5 °C and was gassed with CO_2_ (5%) and O_2_ (95%). Heart was supplied by pacing electrodes connected to the right atrium (RA) appendage to maintain physiological heart rhythm. Local blood supply was induced by 30 min occlusion of the left anterior descending (LAD) coronary artery. The LAD was surrounded by a string at 0.5 cm below the atrioventricular groove, and a small plastic rod was positioned between the heart and the string to ensure the closure of the coronary artery. Thereafter, the heart was reperfused for 30 min. The flow of the perfusion buffer used a “Statham pressure transducer” (P23 Db). A constant preload was kept at 6 mmHg during acclimatization of the heart on the Langendorff system and the perfusion pressure (PP) was kept constant at 50 mmHg during the experimental procedure. Constant PP was secured digitally by means of the perfusion assembly, Module PPCM type 671 (Hugo Sachs Elektronik-Harvard Apparatus GmbH, March, Germany”)).

### 4.2. Induction of Diabetes

Diabetes was induced by a single intraperitoneal injection of 55 mg/kg body weight STZ as described previously [40].

### 4.3. Study Protocol and Study Groups

**Figure 9:**
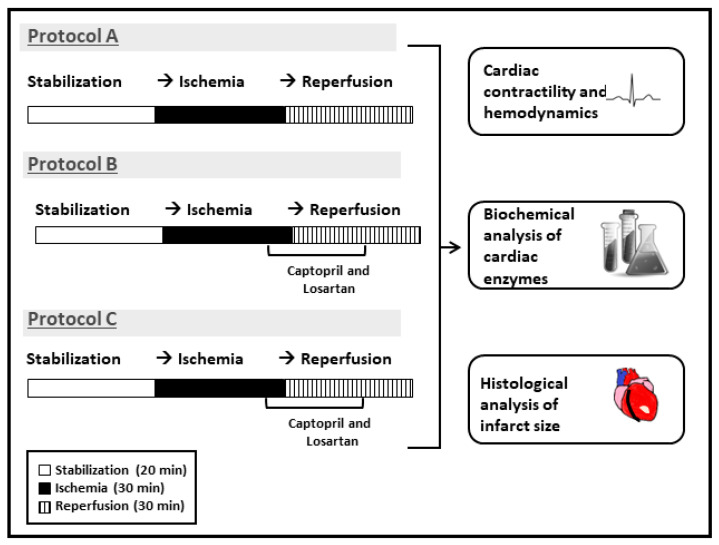
Illustrative drawing showing the study protocols. Hearts isolated from Wistar rats were assigned to one of these protocols (*n* = 8): A, untreated control; B, hearts isolated from rats and subjected to hyperglycemia and treated with Captopril or Losartan or their combination at reperfusion. C, hearts isolated from rats subjected to diabetes for four or six weeks and treated with Captopril or Losartan or their combination at reperfusion.

A total of 96 rats were subdivided into 3 groups (*n* = 32 per group) and subjected to 4 different experimental protocols. The first group contained nondiabetic rats. All hearts isolated from these animals in this group were subdivided into 4 subgroups subjected to acute hyperglycemia which was created by adding 6 g/L to the perfusion buffer. One subgroup (Ctr) was subjected to only I/R and served as control. The second subgroup (Cap) was subjected to I/R and treated with Captopril (100 μM; cat. #:C4042 Sigma-Aldrich (St Louis, MI, USA)). The third subgroup (Los) was subjected to I/R and treated with Losartan (4.5 μM, Santa Cruz Biotechnology). The fourth subgroup (Cap + Los) was subjected to I/R and treated with Losartan (4.5 μM) and Captopril (100 μM). All drugs were injected 5 min before the end of ischemia and continued for 10 min thereafter (Figure 9). The second group was subjected to DM for four weeks, then divided into four subgroups, Ctr, Cap, Los, and Los + Cap (Figure 9). The third group was subjected to DM for six weeks, then like the previous groups, was subdivided into four similar subgroups (Ctr, Cap, Los, and Cap + Los (Figure 9).

### 4.4. Data Collection and Processing

The function of LV was evaluated by the assessment of LV end diastolic pressure (LVEDP), LV maximum developed pressure (DPmax), and LV contractility index (±dP/dt). The coronary–vascular dynamics were assessed by the measurement of the coronary flow (CF) and coronary vascular resistance (CVR). Cardiac hemodynamics were evaluated as described in the previously [60]. Briefly, a latex balloon filled with water was fixed in the LV cavity and connected to a “DC-Bridge amplifier (DC-BA)” of the pressure module (DC-BA type 660, Hugo-Sachs Electronik, Germany) which was connected to a computer for online determination of DPmax. Left ventricular developed pressure was calculated from the acquired values of the “LVESP using Max-Min module Number MMM type 668” (Hugo Sachs Elektronik-Harvard ApparatusGmbH, Germany).

Coronary flow was estimated by electromagnetic flow probe attached to the inflow of the aortic cannula and connected to a computer. This was done digitally and was confirmed manually. The CVR and hemodynamics data were acquired every 10 sec using an “online data acquisition program” (Isoheart software V 1.524-S, Hugo-Sachs Electronik, Germany).

### 4.5. Sample Collection and Storage

Perfusion buffer sample was collected immediately below the cannulated heart from the coronary outflow a few minutes before the end of the reperfusion. The hearts were also collected at the end of the experiments. All samples were frozen in liquid nitrogen and stored at −80 °C for the required biochemical analysis.

### 4.6. Infarct Size Evaluation

The infarct size was evaluated as described previously [60]. Briefly, at the end of experiments the hearts were stored at −20 °C to the next day. Thereafter, the hearts were sliced into thin sections. The sections were then incubated in 1% 2,3,5-Triphenyl-2H-tetrazolium chloride (TTC) solution and then fixed in 4% formaldehyde. The infarcted area and the rest of the LV are located manually on the image. The infarct size was calculated as a percentage of the LV area for every heart. The calculation of the LV area and the infarct size was done using ImageJ (Image J, Wayne Rasb and National Institute of Health, Bethesda, Maryland, USA). Cardiomyocyte injury was confirmed by measuring troponin T (TnT) release in the coronary effluent during the reperfusion period using TnT immunoassay as suggested by the manufacturer’s assay protocols.

### 4.7. Protein Extraction from the Hearts

The hearts were snap-frozen in liquid nitrogen and thereafter stored at −80 °C for biochemical analysis. The left ventricle was homogenized in a lysis buffer (MOPS, 20 mM; KCl, 150 mM; Mg Acetate, 4.5 mM; Triton X, 1%). Protease inhibitor cocktail (Roche Applied Science, Mannheim, Germany, Cat#05892970001) was added to the buffer. The whole mixture was centrifuged at 12,000× g for 15 min at 4 °C. The supernatant was then aliquoted and stored for biochemical analysis. Protein levels were measured using bicinchoninic acid protein assay (Pierce Chemical Co., Rockford, IL, USA, Cat# 23227).

### 4.8. Western Blot Analysis

The frozen protein samples were used in Western blot analysis to evaluate the protein expression using: anti-superoxide dismutase [Cu-Zn] (SOD) antibody (cat# 07-403-I EMD Millipore, Sigma, St. Louis, Missouri, USA), anti-catalase (CAT) antibody (Cat# 12980); anti-extracellular regulated kinases 1/2 (ERK1/2) antibody (cat# 9102); anti-phosphorylated ERK1/2 antibody (cat #9743); anti-caspase-3 antibody (cat #9662); anti-caspase-8 antibody (cat #4927); anti-phosphorylated endothelial nitric oxide synthase (eNOS) (Ser1177) antibody (cat #9571); anti-eNOS (49G3) antibody (cat #Ab #9586) (all from Cell Signaling Technology, Danvers, Massachusetts, USA) and anti-glucose transporter 1 (GLUT-1) antibody (cat #ab115730, Abcam, Cambridge, UK), anti-glucose transporter 4 (GLUT-4) antibody (phosphor S488) (cat #ab188317 Abcam) by standard immunoblot procedures as previously described [61]. Protein extracts were normalized with the lysis buffer to the desired protein concentration. Equal concentrations of protein per lane were calculated for all the samples. The protein band density was corrected to the total loaded protein. After incubation with secondary antibody, the detection was done with the enhanced chemiluminescence technique. A computerized image-acquisition system was used for densitometry analysis.

### 4.9. Estimation of the Inflammatory Cytokines

The aliquoted LV protein (*n* = 4) was used to evaluate the expression of the pro-inflammatory and anti-inflammatory cytokines: tumor necrosis factor alpha (TNF-α) (cat# MBS175904), interleukin 1 beta (IL-1β) (cat# MBS825017), interleukin 6 (IL-6) (cat# MBS355410), and IL-10 (cat# MBS355232) using enzyme-linked immunosorbent assay (ELISA). The protein content was according to the manufacturer’s assay protocols (Biosource International, Camarillo, California USA).

### 4.10. Statistical Analysis

All data were represented as mean ± S.E.M. For analyzing the data, two-way analysis of variance (ANOVA), was performed on absolute values even when the data are presented as % of baseline. For further confirmation of a statistically significant difference, post-hoc analysis with Tukey’s correction was used. Student’s T-Test was used to assess the significance in molecular experiments, infarct size, pre-inflammatory, and anti-inflammatory cytokines (Microsoft Excel). In all cases, *p* < 0.05 was considered statistically significant.

## 5. Conclusions

A blockade of the RAS system protected the diabetic heart from I/R injury. This protection followed a pathway that decreases the apoptosis markers, pro-inflammatory cytokines, and increases the anti-inflammatory cytokines. This protection seems to employ GLUT-4 and a pathway which is not involving ERK1/2 and eNOS.

## Figures and Tables

**Table 1:** Effects of ischemia/reperfusion, diabetes mellitus, and RAS antagonism on the heart contractility (*n* = 8 per group). * *p* < 0.05 compared to respective controls and † *p* < 0.05 compared to the respective ischemic period.

	+dP/dt		-dP/dt	
Treatment	Ischemia	Reperfusion	Ischemia	Reperfusion
Hyperglycemia				
Ctr	55.309 ± 6.23	56.81 ± 4.68	56.81 ± 4.68	58.86 ± 6.31
Ctr + Captopril	54.18 ± 13.00	86.38 ± 3.29 *†	58.61 ± 6.78	95.50 ± 8.82 *†
Ctr + Losartan	55.58 ± 2.91	87.20 ± 7.40 *†	56.93 ± 1.85	90.67 ± 8.29 *†
Ctr + Captopril and Losartan	53.40 ± 4.88	75.88 ± 4.12 *†	58.57 ± 4.06	94.17 ± 9.91 *†
Four weeks diabetic heart				
Ctr	50.32 ± 3.32	48.73 ± 4.56	54.81 ± 3.97	55.05 ± 3.76
Ctr + Captopril	53.29 ± 4.13	87.16 ± 5.31 *†	55.51 ± 4.24	78.57 ± 8.09 *†
Ctr + Losartan	54.68 ± 5.55	70.69 ± 4.33 *†	57.12 ± 3.87	83.99 ± 5.46 *†
Ctr + Captopril and Losartan	53.08 ± 5.68	88.96 ± 3.82 *†	48.89 ± 5.78	74.11 ± 5.35 *†
Six weeks diabetic heart				
Ctr	51.95 ± 3.61	54.83 ± 6.54	50.60 ± 1.16	52.04 ± 2.77
Ctr + Captopril	54.71 ± 4.36	89.28 ± 5.40 *†	50.24 ± 4.22	81.70 ± 8.32 *†
Ctr + Losartan	50.87 ± 6.59	94.64 ± 6.37 *†	54.64 ± 6.37	82.61 ± 7.46 *†
Ctr + Captopril and Losartan	48.60 ± 4.42	80.52 ± 2.12 *†	53.43 ± 2.65	82.18 ± 5.49 *†

**Table 2:** Effects of ischemia/reperfusion, diabetes mellitus, and RAS antagonism on TnT levels (*n* = 4 per group).

Treatment	Troponin C (IU/L)	*p* Value
Hyperglycemia		
Ctr	0.45 ± 0.06	-
Ctr + Captopril	0.15 ± 0.02	0.01
Ctr + Losartan	0.15 ± 0.02	0.01
Ctr + Captopril and Losartan	0.18 ± 0.04	0.01
Four weeks diabetic		
Ctr	0.43 ± 0.03	-
Ctr + Captopril	0.07 ± 0.003	0.001
Ctr + Losartan	0.11 ± 0.02	0.001
Ctr + Captopril and Losartan	0.14 ± 0.03	0.001
Six weeks diabetic	0.27 ± 0.003	-
Ctr	0.14 ± 0.04	0.01
Ctr + Captopril	0.05 ± 0.01	0.001
Ctr + Losartan	0.18 ± 0.03	0.01
Ctr + Captopril and Losartan		

## Data Availability

Data is contained within the article.
